# Dynamic DNA methylation of ovaries during pubertal transition in gilts

**DOI:** 10.1186/s12864-019-5884-x

**Published:** 2019-06-20

**Authors:** Xiaolong Yuan, Shaopan Ye, Zitao Chen, Xiangchun Pan, Shuwen Huang, Zhonghui Li, Yuyi Zhong, Ning Gao, Hao Zhang, Jiaqi Li, Zhe Zhang

**Affiliations:** 1 National Engineering Research Center for Breeding Swine Industry, Guangdong Provincial Key Lab of Agro-Animal Genomics and Molecular Breeding, College of Animal Science, South China Agricultural University, Guangzhou, Guangdong China; 2 State Key Laboratory of Biocontrol, School of Life Sciences, Sun Yat-sen University, North Third Road, Guangzhou Higher Education Mega Center, Guangzhou, 510006 Guangdong China

**Keywords:** Genome-wide DNA methylation, *DNMTs*, Ovaries, Pubertal transition, Gilts

## Abstract

**Background:**

In female mammals, the initiation of puberty, coupling with the dramatically morphological changes in ovaries, indicates the sexual and follicular maturation. Previous studies have suggested that the disrupted DNA methylation results in the delayed puberty. However, to date, the changes in ovarian methylomes during pubertal transition have not been investigated. In this study, using gilts as a pubertal model, the genome-wide DNA methylation were profiled to explore their dynamics during pubertal transition across Pre-, In- and Post-puberty.

**Results:**

During pubertal transition, the follicles underwent maturation and luteinization, coupled with the significant changes in the mRNA expression of *DNMT1* and *DNMT3a*. DNA methylation levels of In-puberty were higher than that of Pre- and Post-puberty at the locations of genes and CpG islands (CGIs). Analysis of the DNA methylation changes identified 12,313, 20,960 and 17,694 differentially methylated CpGs (DMCs) for the comparisons of Pre- vs. In-, In vs. Post-, and Pre- vs. Post-puberty, respectively. Moreover, the CGIs, upstream and exonic regions showed a significant underrepresentation of DMCs, but the CGI shores, CGI shelves, intronic, downstream and intergenic regions showed a significant overrepresentation of DMCs. Furthermore, biological functions of these methylation changes enriched in PI3K-Akt signaling pathway, GnRH signaling pathway, and Insulin secretion, and the mRNA expressions of several genes of these signaling pathway, including *MMP2*, *ESR1*, *GSK3B*, *FGF21*, *IGF1R*, and *TAC3*, were significantly changed across Pre-, In- and Post-puberty in ovaries.

**Conclusions:**

During pubertal transition in gilts, the DNA methylation changes of ovaries were likely to affect the transcription of genes related to PI3K-Akt signaling pathway, GnRH signaling pathway, and Insulin secretion. These observations can provide new insight into the epigenetic mechanism of follicular and sexual maturation during pubertal transition in mammals.

**Electronic supplementary material:**

The online version of this article (10.1186/s12864-019-5884-x) contains supplementary material, which is available to authorized users.

## Background

In humans, puberty is described as a transition from childhood to adulthood [1]. Emerging evidences suggest that pubertal stages and processes show strongly influence on the exhibitions of sexual characteristics [2], reproductive competence [3], cognitive [4], social and emotional development [5]. Much evidence has suggested that the early or delayed puberty appears to have harmful effects on adult health outcomes [6, 7]. However, the mechanism for the pubertal transition is still unclear. In female mammals, the initiation of puberty indicates the sexual and ovarian maturation with the acquisition of the capacity to undergo fertilization and propagate the species [8–10]. Accumulating studies supports the regulatory roles of DNA methylation in folliculogenesis [11]. Moreover, disrupted DNA methylation results in the delayed puberty [12, 13]. Nevertheless, few investigations have focused on the dynamics and changes of DNA methylation in ovaries during the pubertal transition in mammals.

In mammals, the majority of DNA methylation refers to 5mC and is predominantly found in CpG sites [14], which is catalyzed by DNA methyltransferases (DNMTs) using S-adenosyl-methionine as the methyl donor. It is widely deemed that *DNMT1* maintains DNA methylation, while *DNMT3a* and *DNMT3b* are responsible for de novo methylation [14, 15]. Previous studies suggest that CpGs within promoters are more likely to show hypomethylation [16, 17], whereas CpGs in gene bodies are more likely to show hypermethylation [16, 18]. Both hypo- and hypermethylation are closely associated with transcription activities [19]. Importantly, high CpG content promoters (HCPs) and low CpG content promoters (LCPs), showing essentially differentially methylated and expression patterns [20, 21], are strongly associated with housekeeping genes and tissue-specific genes [22, 23], respectively. Additionally, CpG islands (CGIs) are frequently identified as the potential promoters [24] and the regulatory element [25] to support the transcription of genes, and the methylation of CGIs is closely interacted with the methylation of genes [16].

In pigs, puberty is defined as the emergence of the first estrous and follicular maturation [26], and early puberty is selected to shorten the generation interval [27, 28]. Moreover, pigs are the valuable biomedical and pubertal model due to the similar processes in follicular maturation [9, 10] and pubertal transition to humans [29, 30]. In this study, to investigate the dynamic DNA methylation in ovaries during the pubertal transition, using pigs as a pubertal model, ovarian methylomes were acquired from the Pre-, In- and Post-puberty (see Materials and Methods). First, the mRNA expression patterns of *DNMTs* were detected in these pubertal ovaries. Then the dynamics of these ovarian methylomes were profiled for HCP genes, LCP genes, and differentially genic CGIs by using reduced representation bisulfite sequencing (RRBS). Subsequently, these methylation dynamics were explored to determine the biological functions during the pubertal transition. This work can provide useful information into the epigenetic mechanism of follicular and sexual maturation during the pubertal transition for humans.

## Results

### Expression patterns of *DNMT* mRNA in ovarian tissues during pubertal transition

**Fig. 1:**
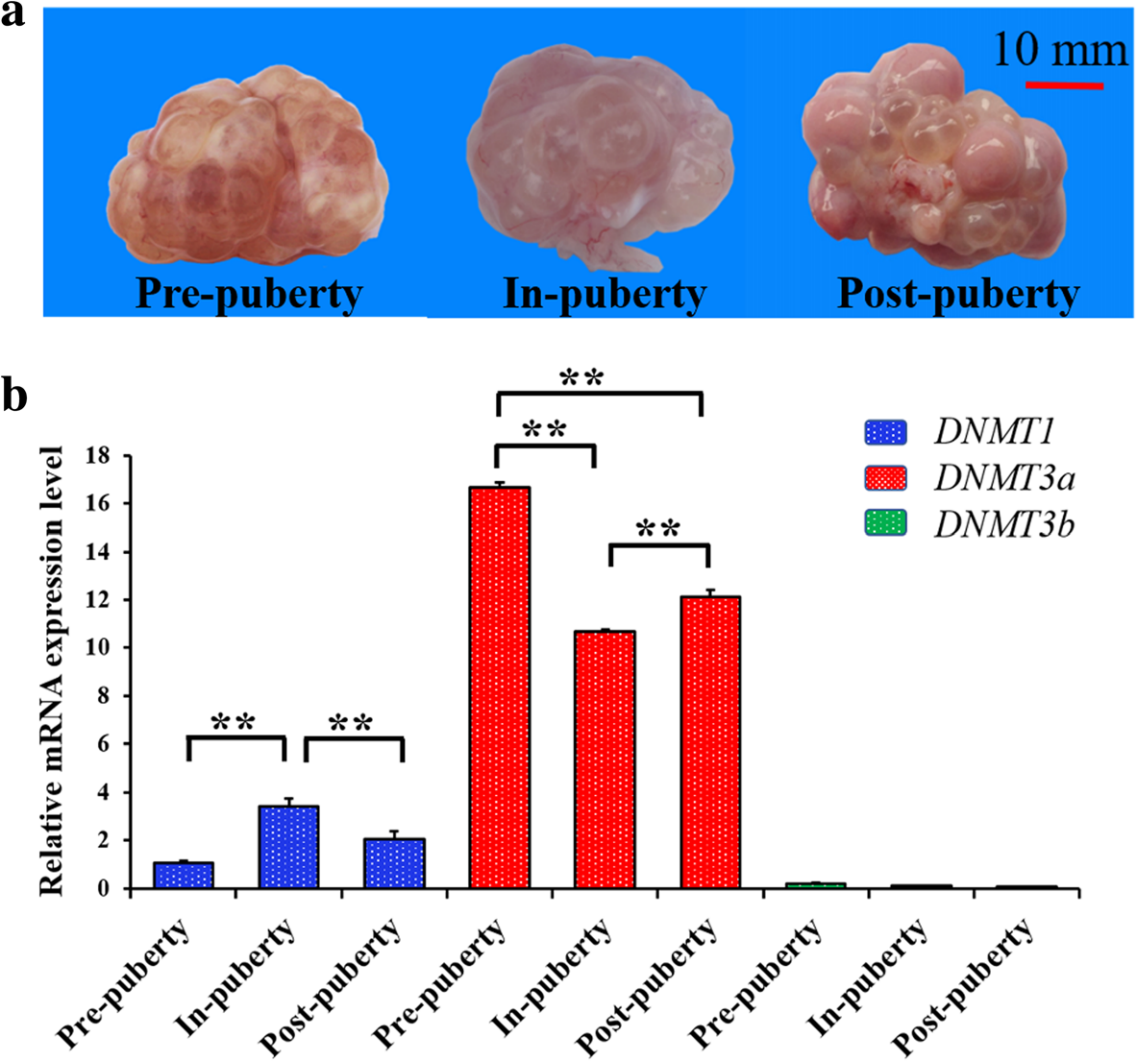
Expression patterns of *DNMTs* mRNA during the pubertal transition. Morphology changes (**a**) and mRNA levels of *DNMTs* (**b**) in ovaries across the Pre-, In- and Post-pubertal stages. Data were expressed as means ± standard error of biological replicates. The significance of differences in means between two groups was analyzed by using Student’s t-test (two-tailed). * indicates *P* < 0.05; ** indicates *P* < 0.01

To explore the DNA methylation changes during the pubertal transition, we first detected the mRNA expression levels of *DNMT1*, *DNMT3a* and *DNMT3b* across the Pre-, In- and Post-pubertal ovaries (Fig. 1). The ovaries and follicles were expected to mature from Pre- to In-puberty (Fig. 1a), and the mature follicles were expected to release oocytes and undergo luteinization from In- to Post-puberty (Fig. 1a). The mRNA levels of *DNMT3a* were higher than those of *DNMT1* and *DNMT3b*, and *DNMT3b* was expressed at the undetectable level. Moreover, the comparisons of these pubertal ovaries uncovered that the mRNA of *DNMT1* was expressed at the highest level at In-puberty (Fig. 1b), but *DNMT3a* was expressed at the lowest level at In-puberty (Fig. 1b). These observations indicated that the maintenance and de novo of DNA methylation might cooperate to initiate ovarian maturation.

### DNA methylation levels of ovarian tissues during pubertal transition

**Fig. 2:**
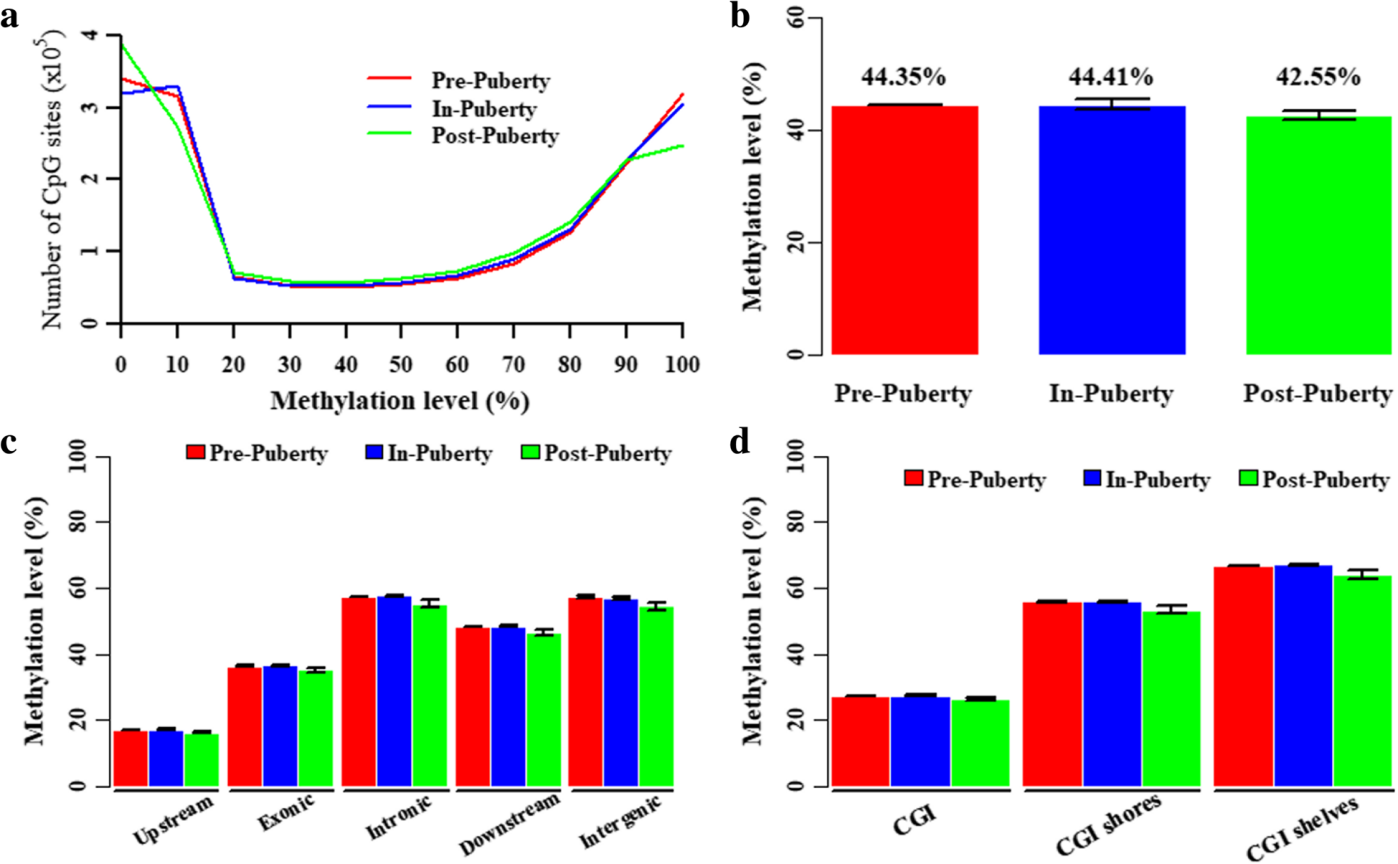
DNA methylation levels of ovarian methylomes during the pubertal transition. (**a**) Distribution of the methylation levels in CpGs. Average methylation levels of CpGs in ovarian methylomes (**b**), CGI-related regions (**c**) and gene-related regions (**d**). Data were expressed as means ± standard error of biological replicates. The significance of differences in means between two groups was analyzed by using Student’s t-test (two-tailed)

Three ovarian methylomes were generated for each pubertal stage. In total, 1,698,083 CpGs covered by at least five reads that coexisted across all nine ovarian tissues remained for further analysis. For each pubertal stage, the methylation level of each CpG site was calculated by the average methylation level across three samples of one group of ovaries. The DNA methylation levels of 1,698,083 CpGs presented a bimodal distribution in Pre-, In- and Post-puberty ovaries, but the distributed features could be clearly distinguished from each other at the hyper-methylated peaks (Fig. 2a). The comparisons among these ovarian methylomes revealed that Post-puberty exhibited the most CpGs with methylation levels less than 20% (Pre-: 43.02 ± 0.39%, In-: 42.37 ± 0.44%, Post: 44.31 ± 0.73%) and CpGs ranging from 50 to 80% (Pre-: 15.65 ± 0.32%, In-: 16.22 ± 0.27%, Post-: 16.97 ± 0.54%), but showed the lowest CpGs with methylation levels higher than 80% (Pre-: 32.50 ± 0.23%, In-: 32.16 ± 0.33%, Post-: 29.53 ± 1.16%) (Fig. 2a). The average methylation levels of the ovarian methylomes were 44.35 ± 0.06%, 44.41 ± 0.22%, and 42.55 ± 0.83% in Pre-, In-, and Post-puberty (Student’s t-test, *P* > 0.05), respectively (Fig. 2b and Table 1), and the average methylation levels of In-puberty were higher than that of Pre- and Post-puberty in the gene-related and CGI-related regions (Fig. 2c,d and Table 1). Additionally, the average methylation levels of the CpGs located in upstream and CGI regions showed the lowest methylated levels, compared with those in the other gene- or CGI-related regions (Fig. 2c,d), although the changes were insignificant (Student’s t-test, *P* > 0.05).

**Table 1:** Average methylation levels of gene-related and CGI-related regions

	Pre-puberty	In-puberty	Post-puberty
Genomes	44.35 ± 0.06%	44.41 ± 0.22%	42.55 ± 0.83%
CGI	27.31 ± 0.10%	27.43 ± 0.21%	26.31 ± 0.54%
CGI shores	55.70 ± 0.03%	55.81 ± 0.21%	53.36 ± 1.07%
CGI shelves	66.77 ± 0.02%	66.95 ± 0.20%	64.05 ± 1.26%
Upstream	16.98 ± 0.06%	17.06 ± 0.12%	16.27 ± 0.40%
intron	57.43 ± 0.05%	57.50 ± 0.19%	55.20 ± 1.05%
exon	36.43 ± 0.12%	36.61 ± 0.24%	35.14 ± 0.67%
Downstream	48.21 ± 0.09%	48.47 ± 0.15%	46.40 ± 0.88%
Intergenic	57.16 ± 0.34%	57.01 ± 0.41%	54.46 ± 1.07%

### Genome-wide DNA methylation in ovarian methylomes

**Fig. 3:**
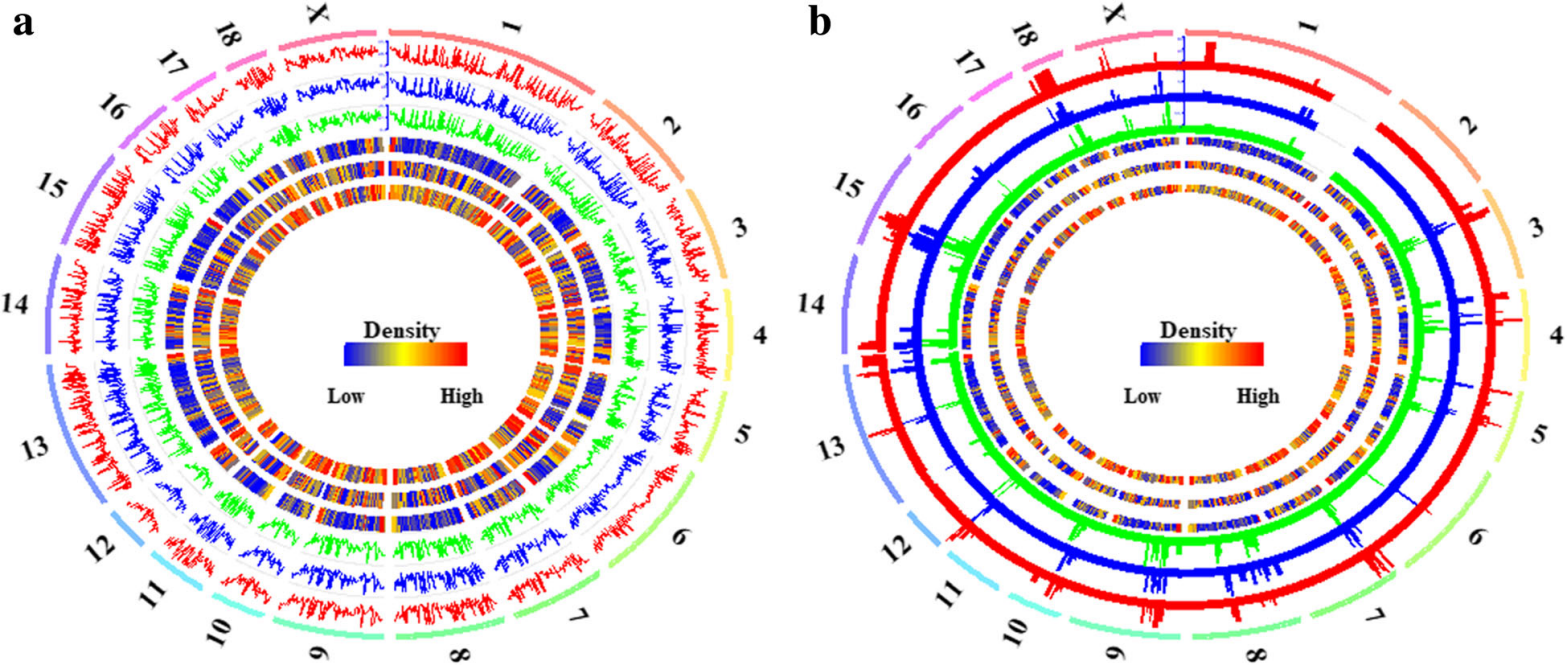
Correlations between these ovarian methylomes and the densities of CpGs, genes and CGIs. (**a**) The global CpG methylation levels in Pre- (track 1), In- (track 2) and Post- (track 3) pubertal methylomes, from outside to inside, were quantified in per 1 Mb window. (**b**) The numbers of the differentially methylated CpG sites (DMCs) between Pre- vs. In- (track 1), In- vs. Post (track 2) and Pre- vs. Post (track 3), from outside to inside, were quantified per 1 Mb window. The densities of the CpGs (track 4), genes (track 5) and CGIs (track 6) in **a** and **b** were also quantified per 1 Mb window. The labels outside of track 1 represent the chromosomes in the pig genome. The methylation levels and density of DMCs were normalized by the number of CpGs in each window

The Pearson’s correlations between the ovarian methylomes and the density of CpGs, genes and CGIs per 1 Mb window were explored and shown in Fig. 3a. Among the ovarian methylomes of Pre-, In- and Post-puberty, the Pearson’s correlation coefficients were all 0.99 (*P* < 2.22 × 10^− 16^), and these ovarian methylomes were positively correlated with the densities of the CGIs (0.26, *P* < 2.22 × 10^− 16^; 0.30, *P* < 2.22 × 10^− 16^; 0.30, *P* < 2.22 × 10^− 16^ in the Pre-, In-and Post-pubertal methylomes, respectively), but negatively correlated with the densities of the genes (− 0.15, *P* < 2.22 × 10^− 16^; − 0.14, *P* < 2.22 × 10^− 16^; − 0.14, *P* < 2.22 × 10^− 16^ in the Pre-, In-and Post-pubertal methylomes, respectively). Additionally, these ovarian methylomes were weakly positively correlated with the density of the CpGs (0.16, *P* < 2.22 × 10^− 16^; 0.18, *P* < 2.22 × 10^− 16^; 0.17, *P* < 2.22 × 10^− 16^ in the Pre-, In-and Post-pubertal methylomes, respectively).

### DNA methylation levels in gene and CGI locations

**Fig. 4:**
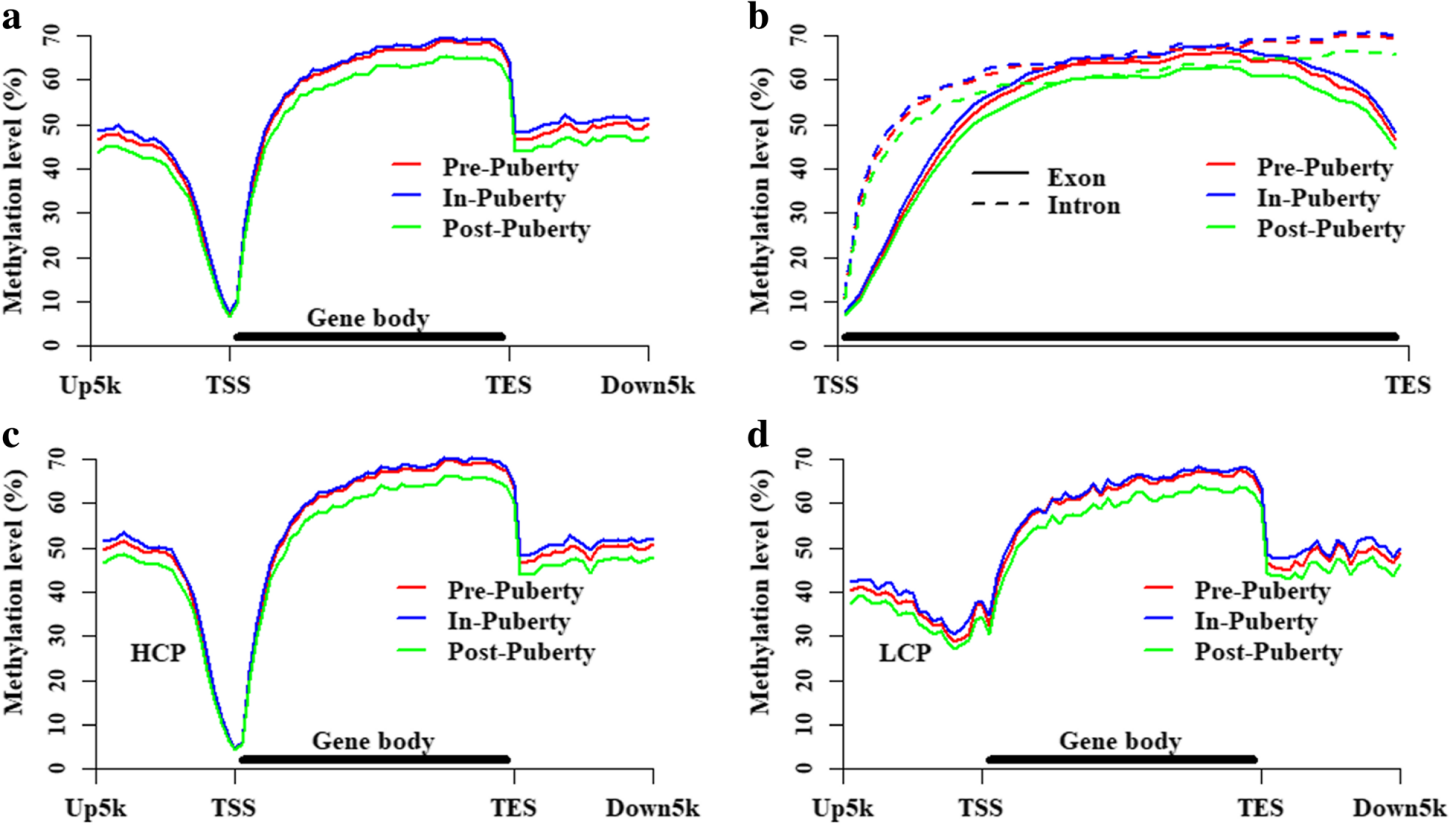
Methylation changes at gene locations in ovarian methylomes. Methylation patterns at the locations of genes (**a**), intronic regions (**b**), exonic regions (**b**), HCP genes (**c**) and LCP genes (**d**). Up5k is denoted as the 5 kb upstream regions of transcription start sites. Down5k is denoted as the 5 kb downstream regions of transcription end sites

To investigative the DNA methylation changes of genic regions in these ovarian methylomes, the methylation patterns of all genes, HCP genes, and LCP genes were profiled in Fig. 4. We found that the methylation levels were lowest at the transcription start sites (TSSs) of the gene locations, and then, the levels increased across the gene bodies but abruptly declined at the transcription end sites (TESs) (Fig. 4a). Additionally, the methylation levels of the introns were higher than those of the exons (Fig. 4b), and the HCP genes (Fig. 4c) displayed differentially methylated patterns from the LCP genes (Fig. 4d), especially at the regions around the TSSs (Fig. 4c,d). As shown in Fig. 4, it was obvious that the DNA methylation levels of In-puberty were higher than that of Pre- and Post-puberty at genic location.

**Fig. 5:**
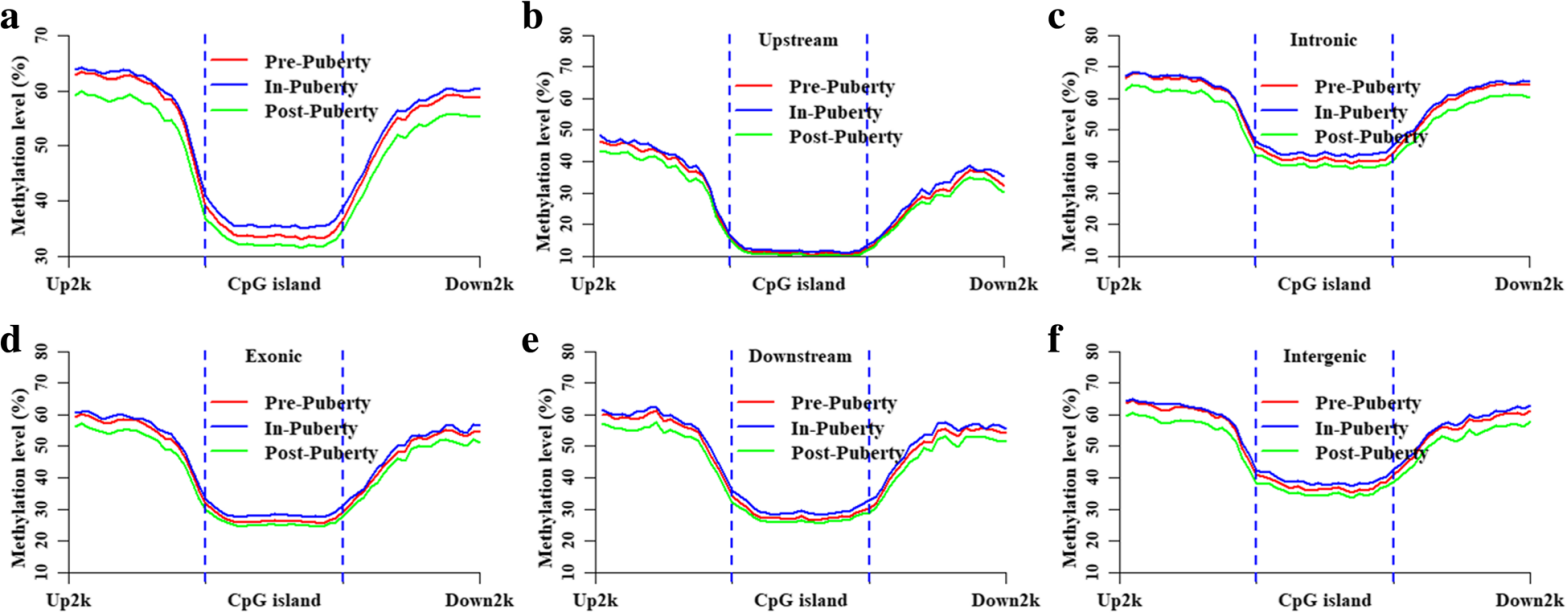
Methylation changes in CGI locations in the ovarian methylomes. Methylated patterns of CGIs located in genomes (**a**), upstream regions (**b**), intronic regions (**c**), exonic regions (**d**), downstream regions (**e**) and intergenic regions (**f**). Up2k is denoted as the 2 kb upstream regions of the CGIs. Down2k is denoted as the 2 kb downstream regions of the CGIs

To determine the changes in the ovarian methylomes at CGI regions, the methylation patterns were profiled in the locations of different genomic CGIs (Fig. 5). At the CGI regions, we found that the methylation level of In-puberty was higher than that of Pre- and Post-puberty (Fig. 5a). These specific CGIs (6250 CGIs) whose average methylation level in Pre- was lower than that in In- but higher than that in Post-puberty comprised 11.27% of Upstream-CGI, 41.41% of Intronic-CGI, 27.01% of Exonic-CGI, 9.82% of Downstream-CGI, and 10.49% of Intergenic-CGI. Totally, 2020 genes that overlapped these specific CGIs were identified, including 1413 HCP and 607 LCP genes. To further determine the biological functions of these specific CGIs, a GO enrichment analysis of these CGI regarding genes was performed. We found that these genes were enriched in Neurogenesis, Neuron differentiation, regulation of transcription from RNA polymerase II promoter (Additional file 1: Figure S1a). The enriched KEGG pathways included the Autophagy-animal, mTOR signaling pathway, Apoptosis, and Insulin resistance (Additional file 1: Figure S1b), which were closely associated with the developments of the ovaries and follicles.

The methylated patterns of the Upstream- (Fig. 5b), Intronic- (Fig. 5c), Exonic- (Fig. 5d), Downstream- (Fig. 5e) and Intergenic-CGIs (Fig. 5f) were profiled to further explore the methylation changes in the ovarian methylomes at the CGI locations. By comparing these CGIs in terms of their different genomic features, we found that the Upstream-CGIs showed the lowest DNA methylation level (Fig. 5b), while Intronic- and intergenic-CGIs showed a relatively higher DNA methylation level (Fig. 5c,f). Moreover, the DNA methylation level could not be separately distinguished among the methylomes in the regions of the Upstream-CGIs (Fig. 5b) but could be separately distinguished in the other genomic CGIs (Fig. 5c-f).

### Changes in ovarian methylomes during pubertal transition

**Fig. 6:**
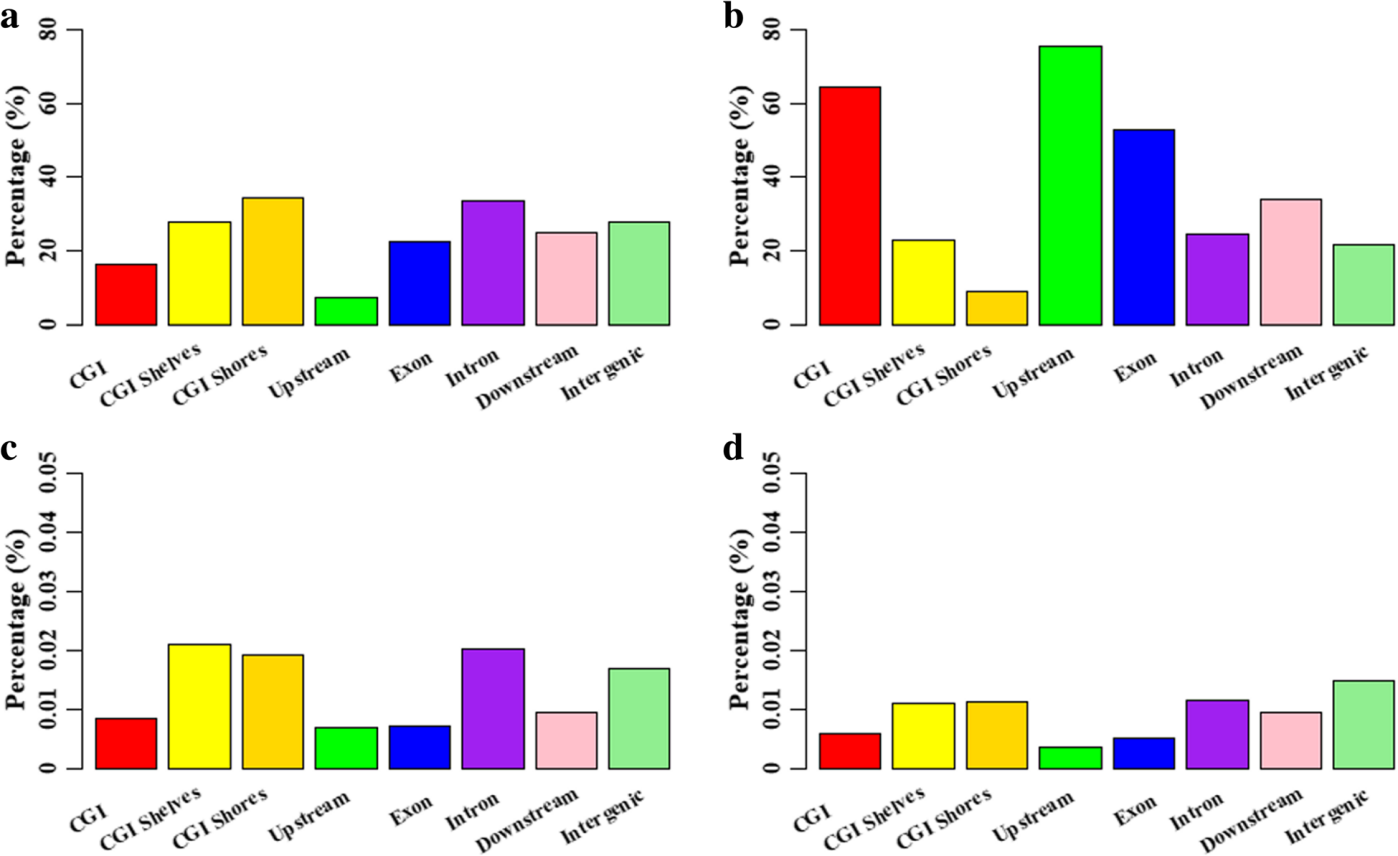
Changes in ovarian methylomes across Pre-, In- and Post-puberty. (**a**) Distribution of consistently hypermethylated CpGs. (**b**) Distribution of consistently hypomethylated CpGs. Distribution of the consistently increasing (**c**) and decreasing (**d**) methylated CpGs

To explore the methylation changes during the pubertal transition in the ovaries, the consistently hypermethylated CpGs (HyperCs) and hypomethylated CpGs (HypoCs) were counted across these ovarian methylomes (Fig. 6a,b). We found that the HypoCs and HyperCs were distributed differentially across the CGI- and gene-related regions (Fig. 6a,b). In total, 16.47 and 7.33% of the detected CpGs located CGI and upstream regions, respectively, were HyperCs, which were lower than those detected in the other gene- and CGI-related regions (Fig. 6a). However, 64.43 and 75.53% of the detected CpGs located in CGIs and upstream regions, respectively, were HypoCs, which were more than those in other gene- and CGI-related regions (Fig. 6b). Interestingly, in the comparisons between the CGIs and upstream regions, the HyperCs were more frequently located at CGIs, but the HypoCs were more likely to locate at upstream regions (Fig. 6a,b). Additionally, 225 consistently increased methylation (IncrmCs) and 154 consistently decreased methylation (DecrmCs) were identified across Pre-, In- and Post-puberty, representing 0.01 and 0.009% of the detected CpGs, respectively (Fig. 6c,d). Both IncrmCs and DecrmCs were more likely to become depleted at the CGI, upstream and exonic regions but overrepresented at the CGI shores, CGI shelves, intronic, downstream and intergenic regions (Fig. 6c,d). Furthermore, the genes related to the IncrmCs were mostly enriched in Notch signaling pathway (Additional file 1: Figure S2a) and the genes related to the DecrmCs were enriched in Necroptosis, Axon guidance, Focal adhesion, GnRH signaling pathway, and NF-kappa B signaling pathway (Additional file 1: Figure S2b).

### Dynamics in ovarian methylomes during pubertal transition

**Fig. 7:**
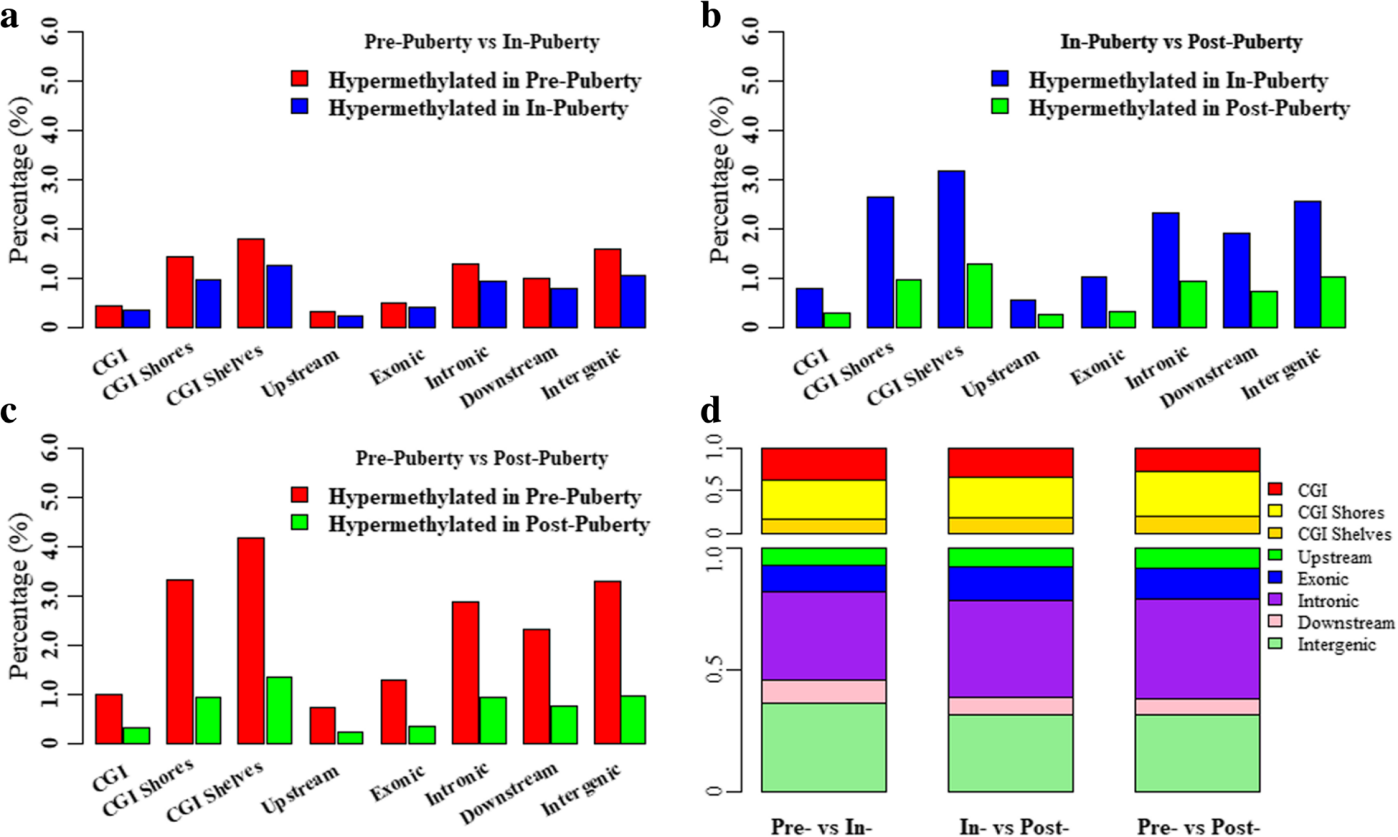
Differentially methylated CpGs and regions among the ovarian methylomes. Distributions of differentially methylated CpG sites between Pre- and In- (**a**), In- and Post- (**b**), and Pre- and Post-puberty (**c**). Distributions of differentially methylated regions among the ovarian methylomes (**d**)

To detect the dynamics of these ovarian methylomes, 840,469 CpGs covered by at least eight reads that coexisted across all nine ovarian tissues were remained to further determine the differentially methylated CpG sites (DMCs). 12,313, 20,960 and 17,694 DMCs, representing 1.47, 2.49 and 2.11% of the detected CpGs with at least eight reads, were identified in Pre- vs. In- (Additional file 2: Table S1), Pre- vs. Post-(Additional file 2: Table S2), and In- vs. Post-puberty (Additional file 2: Table S3), respectively (Fig. 7 and Table 2). We found that DMCs were likely to show a stage-specific distribution among the comparisons of Pre- vs. In-, Pre- vs. Post-, and In- vs. Post- (Fig. 3b). The densities of these DMCs in each group were highly correlated at each chromosome among track 1 vs. track 2 (Pearson’s correlation coefficients = 0.91, *P* < 2.22 × 10^− 16^), track 2 vs. track 3 (Pearson’s correlation coefficients = 0.97, *P* < 2.22 × 10^− 16^), and track 1 vs. track 3 (Pearson’s correlation coefficients = 0.92, *P* < 2.22 × 10^− 16^) (Fig. 3b). The Pearson’s correlation coefficients between the densities of the DMCs and densities of the CpGs were 0.78, 0.83 and 0.83 for Pre- vs. In-, Pre- vs. Post-, and In- vs. Post-, respectively (*P* < 2.22 × 10^− 16^). The densities of the DMCs were highly correlated with the densities of the CGIs for Pre- vs. In- (0.83, *P* < 2.22 × 10^− 16^), Pre- vs. Post- (0.90, *P* < 2.22 × 10^− 16^), and In- vs. Post- (0.89, *P* < 2.22 × 10^− 16^) but were moderately correlated with the densities of genes for Pre- vs. In- (0.20, *P* < 2.22 × 10^− 16^), Pre- vs. Post- (0.24, *P* < 2.22 × 10^− 16^), and In- vs. Post- (0.24, *P* < 2.22 × 10^− 16^).

**Table 2:** Distribution of differentially methylated CpGs among the ovarian methylomes

	Detected CpGs	DMCs between Pre- and In-puberty		DMCs between Pre- and Post-puberty		DMCs between In- and Post-puberty	
		Number	Enrichment	Number	Enrichment	Number	Enrichment
Total	840,469	12,313	–	20,960	–	17,694	–
CGI	501,376	3813	0.30 (*P* < 2.22 × 10^−16^)	6324	0.29 (*P* < 2.22 × 10^− 16^)	5226	0.28 (*P* < 2.22 × 10^− 16^)
CGI shores	138,931	3161	1.74 (*P* < 2.22 × 10^− 16^)	5578	1.83 (*P* < 2.22 × 10^− 16^)	4778	1.87 (*P* < 2.22 × 10^− 16^)
CGI shelves	40,091	1141	2.04 (*P* < 2.22 × 10^− 16^)	2060	2.18 (*P* < 2.22 × 10^− 16^)	1672	2.08 (*P* < 2.22 × 10^− 16^)
Upstream	182,275	959	0.30 (*P* < 2.22 × 10^− 16^)	1678	0.31 (*P* < 2.22 × 10^− 16^)	1373	0.30 (*P* < 2.22 × 10^− 16^)
intron	219,465	4718	1.76 (*P* < 2.22 × 10^− 16^)	8051	1.76 (*P* < 2.22 × 10^− 16^)	6871	1.79 (*P* < 2.22 × 10^− 16^)
exon	214,079	1850	0.52 (*P* < 2.22 × 10^− 16^)	3376	0.56 (*P* < 2.22 × 10^− 16^)	2817	0.55 (*P* < 2.22 × 10^− 16^)
Downstream	46,202	759	1.12 (*P* < 2.22 × 10^− 16^)	1318	1.15 (*P* < 2.22 × 10^− 16^)	1138	1.18 (*P* < 2.22 × 10^− 16^)
Intergenic	178,448	4027	1.80 (*P* < 2.22 × 10^− 16^)	6537	1.68 (*P* < 2.22 × 10^− 16^)	5495	1.67 (*P* < 2.22 × 10^− 16^)

Enrichment of DMCs for certain genomic regions was using a two-tailed Fisher’s exact test

Moreover, these DMCs were differentially distributed in the CGI- and gene-related regions, and were observed to be hypomethylated in Post- but hypermethylated in Pre- and In-puberty (Fig. 7a-c). Among the comparisons of these ovarian methylomes, the CGIs, upstream and exonic regions showed a significant underrepresentation of DMCs (relative enrichment = 0.28–0.56, *P* < 2.22 × 10^− 16^, Table 2), but the CGI shores, CGI shelves, intronic, downstream and intergenic regions showed a significant overrepresentation of DMCs (relative enrichment =1.12–2.18, *P* < 2.22 × 10^− 16^, Table 2). Furthermore, 201, 534 and 353 differentially methylated regions (DMRs) were identified for Pre- vs. In- (Additional file 2: Table S4), Pre- vs. Post- (Additional file 2: Table S5), and In- vs. Post-puberty (Additional file 2: Table S6), respectively (Fig. 7d). DMRs were more likely to occur in CGI, CGI shores, exons, introns and intergenic regions but were likely to be depleted in CGI shelves, upstream and downstream regions (Fig. 7d).

**Fig. 8:**
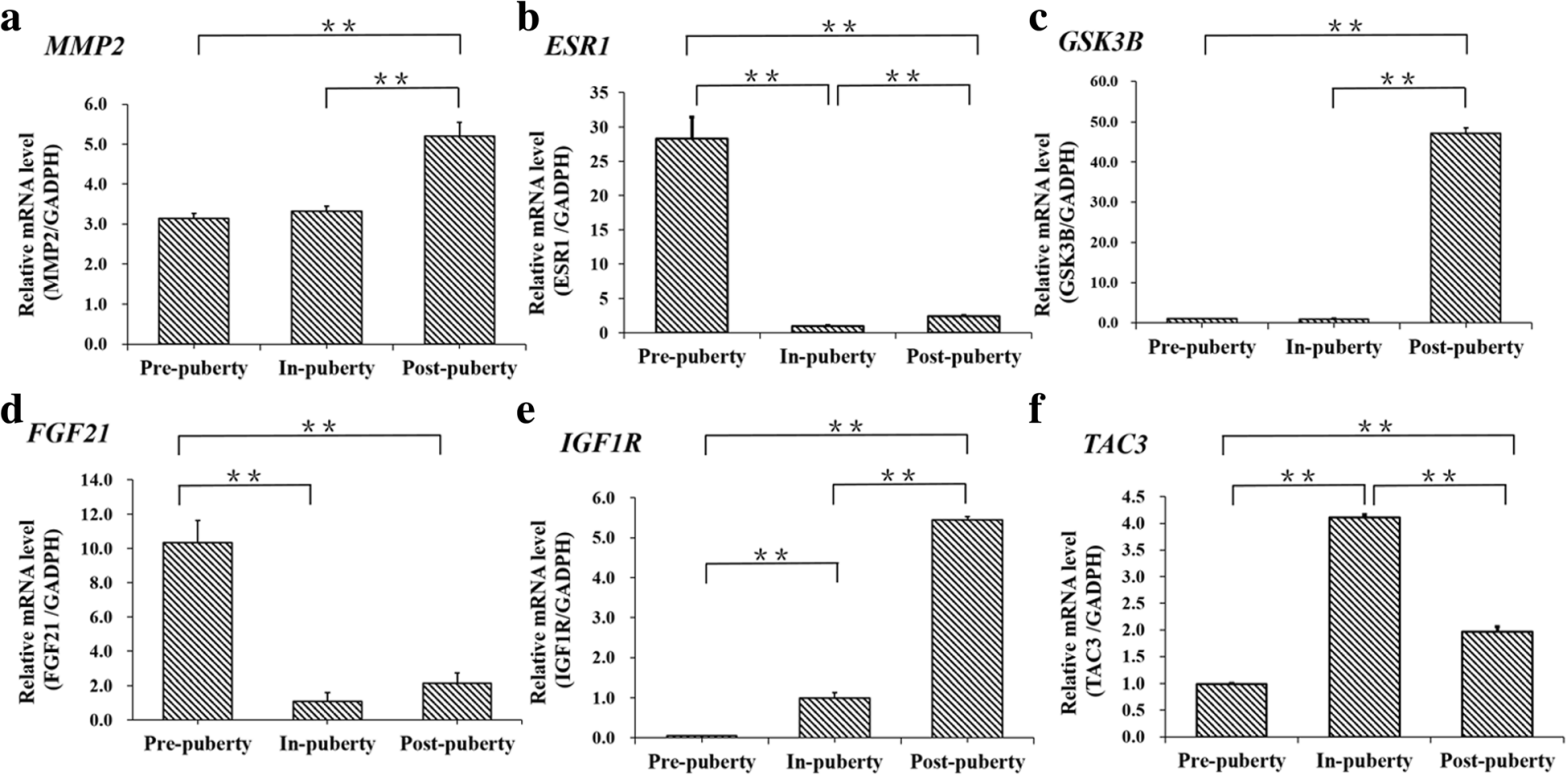
mRNA expression levels of several DMR regarding genes across the pubertal transition in ovaries. The mRNA expression changes of *MMP2* (**a**), *ESR1* (**b**), *GSK3B* (**c**), *FGF21* (**d**), *IGF1R* (**f**), and *TAC3* (**f**) in ovaries during the pubertal transition in gilts. The significance of differences in means between two groups was analyzed by using Student’s t-test (two-tailed). Data were expressed as means ± standard error of biological replicates. ** indicates *P* < 0.01

To gain insight into the biological processes in which the DMR genes might be involved, we performed the KEGG enrichment analysis on the genes exhibiting at least one DMR. In total, 598 genes were related to at least one DMR, and these DMR genes were enriched in the PI3K-Akt signaling pathway, MAPK signaling pathway, mTOR signaling pathway, GnRH signaling pathway, Insulin secretion, cortisol synthesis and secretion (Additional file 1: Figure S3). Moreover, several key genes of PI3K-Akt signaling pathway, GnRH signaling pathway, Insulin secretion, whose upstream regions contained at least one DMC, were selected, and their mRNA expressions were detected across the pubertal transition in ovaries. We found that the mRNA levels of *MMP2* (Fig. 8a), *ESR1* (Fig. 8b), *GSK3B* (Fig. 8c), *FGF21* (Fig. 8d), *IGF1R* (Fig. 8e), and *TAC3* (Fig. 8f) were significantly changed across Pre-, In- and Post-puberty in ovaries.

## Discussions

During pubertal transition in both humans and pigs, the morphologies of ovaries dynamically transformed along with follicular and sexual maturation [9, 10]. To compare and describe the dynamics of methylation, ovarian methylomes of Pre-, In- and Post-pubertal stage were profiled by using RRBS. We found that the average methylation levels of the ovarian methylomes (Fig. 2b), gene- (Fig. 2c) and CGI-related region (Fig. 2d) in In-stage were higher than that in Pre- to Post-stage (Table 1, Student’s t-test, *P* > 0.05), and these methylation changes could be clearly distinguished from each other at the hypo and hyper-methylated peaks (Fig. 2a).

Previous studies have demonstrated that the DNA methylation of HCP genes is likely to be maintained by *DNMT1*, and that LCP genes was likely to be catalyzed by other *DNMTs* for de novo methylation [14, 15]. In the present study, the mRNA expression of *DNMT1* and *DNMT3a* in ovaries significantly changed in the ovaries across the Pre-, In- and Post-stage (Fig. 1), suggesting that DNA methylation might be involved in follicular and sexual maturation. The methylated patterns of gene locations were profiled to investigate the changes in these ovarian methylomes (Fig. 4). We found that the Post-stage displayed the lowest DNA methylation levels, and the DNA methylation level of In-stage was observed to be a little higher than Pre- stage in all genes (Fig. 4a), HCP (Fig. 4c) and LCP (Fig. 4d) gene locations, even at the intron and exon regions (Fig. 4b). Additionally, the methylation levels at the gene locations declined abruptly at TSSs and TESs (Fig. 4a). These observations are consistent with previous studies in humans [31], pigs [16] and mice [32].

The LCP and HCP gene locations showed differentially methylated patterns (Fig. 4c,d), which is in line with others studies [21, 33], and the methylation levels in intronic regions were higher than those in the exonic regions (Fig. 4b), especially around the TSSs and TESs across the gene bodies, which might have be explained by the appearance of the CpG density, which was lower in the introns than that in the exons [34]. Moreover, the average DNA methylation level of the CGIs in the Pre-stage was lower than that in In-stage but higher than that in Post-stage (Fig. 5a). Furthermore, 69.95% of the related genes in these specific CGIs whose methylation level in the Pre- was lower than that in the In- but higher than that in the Post-pubertal stage were HCP genes, and only 30.05% of the related genes in these specific CGIs were LCP genes. Previous studies suggest that HCPs are strongly associated with housekeeping genes [22, 23], and that LCPs are strongly associated with tissue-specific genes [22, 23]. Moreover, the related genes in these specific CGIs were enriched in Neurogenesis, Neuron differentiation, Autophagy-animal, mTOR signaling pathway, Apoptosis, Insulin resistance, and Insulin signaling pathway and (Additional file 1: Figure S1). It is possible that the hormones secreted by ovaries show a strong feedback on the Neuron development during the pubertal transition in gilts.

The HypoCs tended to be found in the CGIs, upstream and exonic regions rather than in other CGI- and gene-related regions (Fig. 6b), which is consistent with previous studies in mammals [16, 35]. The HyperCs were depleted in the CGIs and upstream regions but occurred more frequently in other CGI-and gene-related regions (Fig. 6a). In total, 80.90, 82.86 and 75.65% of the detected CpGs located in CGIs, upstream and exonic regions were HypoCs or HyperCs across these ovarian methylomes (Fig. 6a,b). Moreover, the DecrmCs and IncrmCs were more likely to be depleted in the CGIs, upstream and exonic regions (Fig. 6c,d) rather than the CGI- and gene-related regions.

The DMCs were also depleted in the CGIs, upstream and exonic regions, but occurred more frequently in the other CGI- and gene-related regions (Fig. 7a-c). However, the DMRs were likely to occur in the CGI, CGI shores, introns, exon regions but were depleted from the CGI shelves, upstream, downstream and intergenic regions (Fig. 7d). Furthermore, the genes related to DMRs were highly associated with reproduction developmental processes such as PI3K-Akt signaling pathway, GnRH signaling pathway, and Insulin secretion (Additional file 1: Figure S3). The ovarian mRNA levels of several key genes of these signaling pathways, whose upstream regions contained at least one DMC, were detected during pubertal transition by RT-PCR (Fig. 8). We found that the mRNA of *MMP2* of the GnRH signaling pathway and Estrogen signaling pathway, which displayed a peak at the ovulation-luteogenesis transition phase in pubertal rat [36], expressed the highest in the ovaries of Post-puberty in gilts (Fig. 8a); the mRNA level of *ESR1* of Estrogen signaling pathway, whose loss-of-function mutation caused into the delayed puberty in woman [37], displayed the highest at the Pre-puberty (Fig. 8b); the mRNA level of *GSK3B* of Insulin signaling pathway, which is highly associated with the age of puberty in cattle [38], exhibited the highest level at Post-puberty (Fig. 8c); the mRNA level of *FGF21*, from PI3K-AKt signaling pathway, positively associated with luteinizing hormone [39], a hormone-like circulating protein recently identified as a metabolic regulator of glucose and lipid metabolism [40], expressed the highest at Pre-puberty (Fig. 8d); the mRNA level of *IGF1R*, from Ovarian steroidogenesis pathway, which is essential for steroidogenesis and follicle survival in the ovarian granulosa cells of mice [41], displayed the highest at Post-puberty (Fig. 8e); the mRNA level of *TAC3*, which accelerated follicle development and enhanced estradiol production in the ovaries of zebrafish [42], exhibited the highest at the In-puberty (Fig. 8f). These observations indicated that DNA methylation changes might affect the transcription and expression of genes associated to the follicle development, and thus produce an effect on the ovarian mature during pubertal transition in gilts.

In this study, although the genome-wide DNA methylation patterns of ovaries were clearly depicted during the pubertal transition in gilts, there were two main limitations. The first limitation was that the ovarian DNA methylomes of pubertal transition were acquired from the whole ovarian tissues. The changes of DNA methylation will become clearer upon using specific ovarian cells during pubertal transition. Another limitation was that although RRBS is competent to capture a comprehensive and representative fraction of CpGs throughout the genome, the characterization of ovarian methylomes during pubertal transition will be more complete with more improved coverage of whole genome.

## Conclusions

Collectively, during the pubertal transition in gilts, the ovaries underwent maturation, coupling with the significant changes in the mRNA expression of *DNMT1* and *DNMT3a*. DNA methylation levels of In-puberty were higher than that of Pre- and Post-puberty at the location of genes and CGIs. Moreover, the DNA methylation changes were stage-specific and were likely to affect the transcription of genes related to PI3K-Akt signaling pathway, GnRH signaling pathway, and Insulin secretion that were highly associated with the reproduction developmental processes.

## Methods

### Ethics statement

The pig cares and the experiments were conducted according to the Regulations for the Administration of Affairs Concerning Experimental Animals (Ministry of Science and Technology, China) and were approved by the Animal care and Use Committer of South China Agricultural University, Guangzhou, China (approval number: SCAU#2013–10). The pigs were fed the same diet ad libitum and reared under the same conditions in the same environments. The ovaries were collected, frozen quickly in liquid nitrogen and then stored at − 80 °C for further using.

### Animals and sample preparation

The ovarian samples were collected from Landrace × Yorkshire crossbred gilts bought from Zhongshan Baishi Piggery Co., Ltd. (Zhongshan, China). The onset of pig puberty could be easily identified by the standing reflex with the back-pressure test and boar contact [43]. 25 Landrace × Yorkshire crossbred gilts aged at 160 days were prepared for this study. Pubertal signs were checked and recorded twice daily at 09:00 and 15:30 by inspection of the vulva and assessment of the standing reflex for these 25 gilts. Three pigs aged at 180 days without pubertal signs were selected as Pre-pubertal gilts; three gilts at the day exhibiting the first estrous and the standing reflex were selected as the In-pubertal gilts (about 205 days); and another three gilts in the dioestrus phase, 14 days after the day exhibiting the first estrus and standing reflex, were selected as the Post-pubertal gilts. The gilts were fed the same diet daily and reared in the same conditions and environments. The other 16 pigs were called off. The ovaries of each pubertal group were collected and frozen quickly in liquid nitrogen, ground in a mortar containing liquid nitrogen, and stored at − 80 °C for further using.

### Quantitative real-time PCR

The total RNA was extracted using TRIzol reagent (TaKaRa, Tokyo, Japan) and reverse-transcribed using a RevertAid First Strand cDNA Synthesis Kit (Thermo Scientific, USA) for mRNAs. The relative expression levels of the mRNAs were quantified using Maxima SYBR Green qRT-PCR Master Mix (2×) (Thermo Scientific) and THUNDERBIRD SYBR qPCR Mix (Toyobo) on a LightCycler Real-Time PCR system. The expression levels of *GAPDH* mRNAs were used as endogenous controls, and the fold changes were calculated using the 2^-ΔΔct^ method. The primer sequences are listed in Table 3. The mRNA samples are from the same pigs used for methylation profiles.

**Table 3:** Primers used for RT-PCR in the present study

Name	Sequence	Product(bp)	Accession number
*DNMT1*	F: GGGAGATGTGGAGATGCTATGC	271	NM_001032355.1
	R: TGCTGGTACTGAAGCGGTTG		
*DNMT3a*	F: GCGAAGTGAGGACCATTACTACGA	126	NM_001097437.1
	R: AGCCGAACACCCTTTCCATCT		
*DNMT3b*	F: GAGCGTAGAAGCAAGGAAACAC	332	NM_001348900.1
	R: CTGGCAGCCGTCATCGTCATA		
*ESR1*	F:GATGCCTTGGTCTGGGTGAT R:AGTGTTCCGTGCCCTTGTTA	124	NM_214220.1
*FGF21*	F:CACGAAACTGAAGCCCACCTG R:TTGTAGCCATCCTCAAGAAGC	165	NM_001163410.1
*GSK3B*	F:AACCACCTTCTTTGCGGAG R:GCTTGGCTTGATACACGACA	214	NM_001128443.1
*IGF1R*	F:AGAACTGCACGGTGATCGAG R:AGATGACCAGGGCGTAGTTG	178	NM_214172.1
*TAC3*	R:GACTTCTTTGTGGGTCTTATGG F:GCAGTTTCTACAGACGGTGG	283	NM_001007196.1
*MMP2*	R:GATGGCTTCCTTTGGTGTTCC R:CTTCTTGTCGCTGTCGTAGTCC	318	NM_214192.2
*GAPDH*	F:TCCCGCCAACATCAAAT R:CACGCCCATCACAAACAT	253	NM_001206359.1

### RRBS library and data analysis

The library constructions and sequencing services were provided by RiboBio Co., Ltd. (Guangzhou, China) as previously described in our studies [16, 33, 44]. The genomic DNA of these ovarian tissues was extracted using a DNeasy Blood & Tissue Kit (Qiagen, Beijing), and then, after checking on the quality of the extracted DNA, one library was built for each pigs based on previously published RRBS studies [16, 33, 44]. The processes and procedures of RRBS libraries were briefed as follows. Firstly, the purified genomic DNA was digested overnight with *Msp*I (New England Biolabs, USA). For the *Msp*I digested segments, the sticky ends were filled with CG nucleotides and 3′ A overhangs were added. Secondly, methylated Illumina sequencing adapters with 3′ T overhangs were ligated to the digested segments, and the products obtained were purified. Then 110–220 bp fragments were selected [44] and converted by bisulfite using an EZ DNA Methylation Gold Kit (Zymo Research, USA). Lastly, libraries of 110–220 bp fragments were PCR amplified and each library was sequenced using one lane of an Illumina HiSeq 2500 and 100 bp paired-end reads.

### Bioinformatic of RRBS data

About 30 million paired-end reads were generated for each sample (Additional file 1: Table S7), and the reads mapping to unique locations were about 73% (Additional file 1: Table S7). The first two nucleotides were trimmed from all the second read sequences to blunt-end the *Msp*I site. All reads were trimmed using Trim Galore (v0.4.0) software (Babraham Bioinformatics, http://www.bioinformatics.babraham.ac.uk/projects/trim_galore/) and a Phred quality score of 20 as the minimum. The adaptor pollution reads and multiple N reads (where *N* > 10% of one read) were removed to generate the clean reads. The quality control checks were performed by FastQC (v0.11.3) software (Babraham Bioinformatics). The clean reads were mapped to the pig reference genome (Sscrofa 11.1, downloaded from Ensembl, http://asia.ensembl.org/Sus_scrofa/Info/Index), and then, the DNA methylation calling was performed by Bismark (v0.14.5) [45] using the default parameters. In order to avoid counting the overlapping methylation calls twice for the overlapped reads, only the methylation calls of read 1 were further used with the option —no_overlap by Bismark. The bisulfite conversion rates of these nine samples were all greater 99% (Additional file 1: Table S7).

After the DNA methylation calling by Bismark [45] for these nine RRBS datasets, 1,698,083 CpGs covered by at least five reads that coexisted across all nine ovarian methylomes remained for further analysis. The methylation level of the CpGs was calculated as the methylated reads divided the total covered reads. The methylation level of one group of ovaries was calculated by the average methylation level across the three replicates. For each specific region, the methylation level was measured as the average level of CpGs located in this region. To profile the DNA methylation patterns at the gene and CGI locations, the gene locations were divided into 20, 40 and 20 bins for 5 kb upstream region of the transcription start sites (TSSs), gene body and 5 kb downstream region of transcription end sites (TESs), respectively, and the CGI locations were divided into 20, 20 and 20 bins for 2 kb upstream region, CGIs and 2 kb downstream region, respectively. These analyses were performed by Perl and R scripts. This pipeline was carefully described by our previous studies [16, 33].

The HyperCs were defined as CpGs that were consistent with methylation level ≥ 80% across Pre-, In- and Post-puberty; The HypoCs were denoted as CpGs that were consistent with methylation level ≤ 20% across Pre-, In- and Post-puberty; The IncrmCs were annotated as CpGs whose methylation level increased by ≥ 20% from Pre- to In-puberty and ≥ 20% from In- to Post-puberty; The DecrmCs were annotated as CpGs whose methylation level decreased by ≥ 20% from Pre- to In-puberty and ≥ 20% from In- to Post-puberty.

### Annotation of CGI and gene location

The pig CGI locations were downloaded from UCSC (http://hgdownload.soe.ucsc.edu/goldenPath/susScr11/database/). The CGIs were described as regions > 200 bp in length, with a C and G percentage > 0.5 and a ratio of observed CpG/expected CpG > 0.6. The expected CpGs were calculated as (GC content/2)^2^. The +/− 2 kb regions outside the CGIs were defined as CGI shores, and the +/− 2 kb regions outside the CGI shores were defined as CGI shelves. The gene locations were downloaded from Ensembl (http://asia.ensembl.org/Sus_scrofa/Info/Index). Based on the gene locations in Ensembl, the pig genome was separated into five genic features, including the upstream, exonic, intronic, downstream and intergenic regions. The upstream region was 5 kb upstream region of the TSS. The exon was defined as the integration of the 5′UTR, CDS and 3′UTR arranging from the TSS to the TES. The intron was determined as the integration of introns arranged from the TSS to the TES. The downstream region was defined as the 5 kb downstream region of the TES. The intergenic region was denoted as the outside regions of the upstream, exonic, intronic and downstream regions. When the overlap ration between a CGI and the specific genic feature was greater than 50%, that CGI was classified with the specific genic feature. According to the overlap between a CGI and a specific genic feature, the CGIs were annotated as Upstream-CGI, Intronic-CGI, Exonic-CGI, Downstream-CGI or Intergenic-CGI. The processes and procedures of the CGI annotation have been described in detail in our previous studies [16]. Genes that overlapped a CGI were identified as the CGI regarding genes.

### Statistical analysis

According to our previous study, the porcine genes could be separated into two classes: HCP and LCP genes [33]. The significant differences between two groups were tested using a Student’s t-test with the function of “t.test”, the enrichment was tested using a two-tailed Fisher’s exact test with the function of “fisher.test”, and the Pearson’s correlation coefficient was tested with the function of “cor.test” in R “stats” package (https://www.rdocumentation.org/packages/stats/). * indicates *P* < 0.05; ** indicates *P* < 0.01.

The DMCs and DMRs were calculated by CGmapTools [46]. 840,469 CpGs covered by at least eight reads that coexisted across all nine ovarian tissues remained for further analysis of DMCs. The CpGs whose methylation levels changed more than 20% were identified as DMCs according to a two-tail Fisher’s exact test corrected by the false discovery rate (*P* ≤ 0.05). The DMRs were defined by using the dynamic fragmentation strategy [46] with *P* ≤ 0.05 (corrected by the false discovery rate) and delta methylation ≥ 20%, based on CpGs covered by at least five reads. The enrichment of DMCs in certain genomic regions was calculated by using a two-tail Fisher’s exact test. The genes, including 5 kb upstream flanking region, gene body and 5 kb downstream flanking region, overlapped at least one DMR were defined as the DMR regarding genes. The GO and KEGG enrichment analysis were performed by the R package “clusterProfiler” [47] according to the over-representation test (*P* ≤ 0.05) [48]. The background genes of GO and KEGG enrichment analysis were the genes, including 5 kb upstream flanking region, gene body and 5 kb downstream flanking region, containing as least one detected CpGs.

## Additional files

Additional file 1: The biological function analysis of specific CGIs. (DOCX 2242 kb)

Additional file 2: The summary count tables of RRBS data and genomic coordinates of DMCs and DMRs. (XLSX 2891 kb)

## Data Availability

The RRBS data used in this study have been submitted on European Nucleotide Archive under accession numbers: PRJEB32261.

## Abbreviations

CGIs: CpG islands
DecrmCs: Consistently decreased methylation
DMCs: Differentially methylated CpG sites
DMRs: Differentially methylated regions
DNMTs: DNA methyltransferases
HCPs: High CpG content promoters
HyperCs: Consistently hypermethylated CpGs
HypoCs: Consistently hypomethylated CpGs
IncrmCs: Consistently increased methylation
LCPs: Low CpG content promoters
RRBS: Reduced representation bisulfite sequencing
TESs: Transcription end sites
TSSs: Transcription start sites
