# Supplementary material for: Dynamic DNA methylation of ovaries during pubertal transition in gilts
Source: BMC Genomics. 2019 Jun 20;20:510. doi: 10.1186/s12864-019-5884-x (PMC6585006; doi:10.1186/s12864-019-5884-x)
Supplement: Supplementary file 1 — The biological function analysis of specific CGIs. (DOCX 2242 kb) [file 12864_2019_5884_MOESM1_ESM.docx]

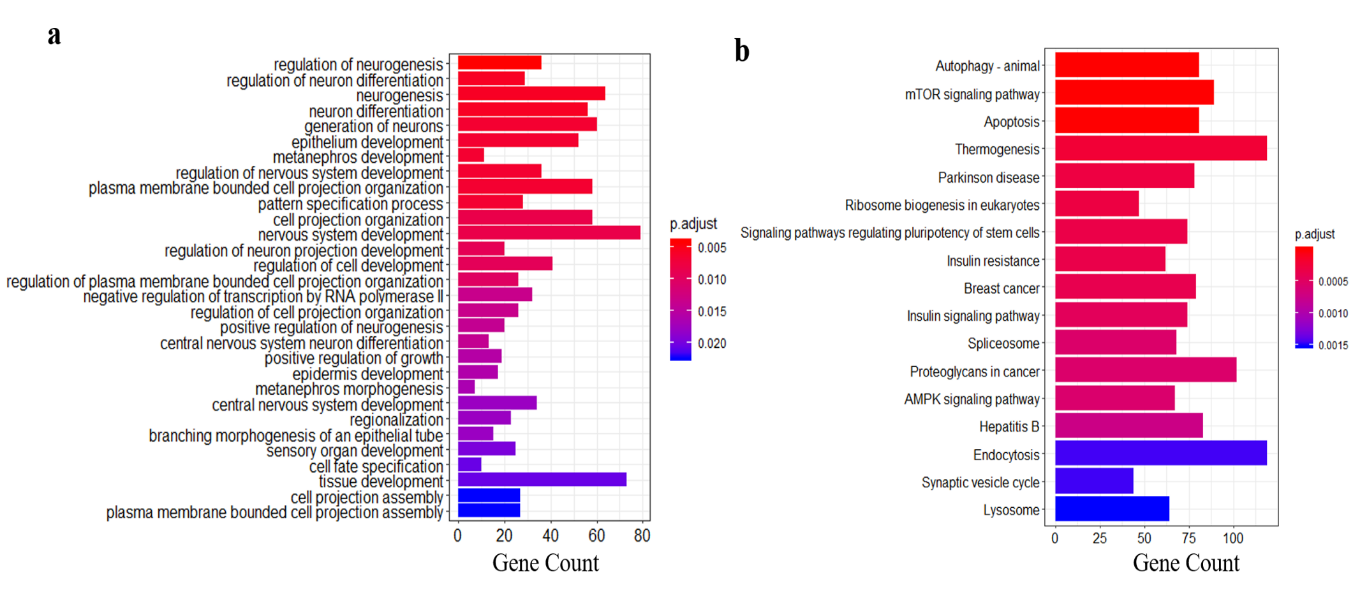


**Figure S1.** **Enriched developmental process in specific CGIs**. (**a**) The enriched biological processes of GO terms. (b) The enriched KEGG pathways. These specific CGIs were defined as CGIs whose methylation level in Pre- was lower than that in In- but higher than that in Post-puberty.


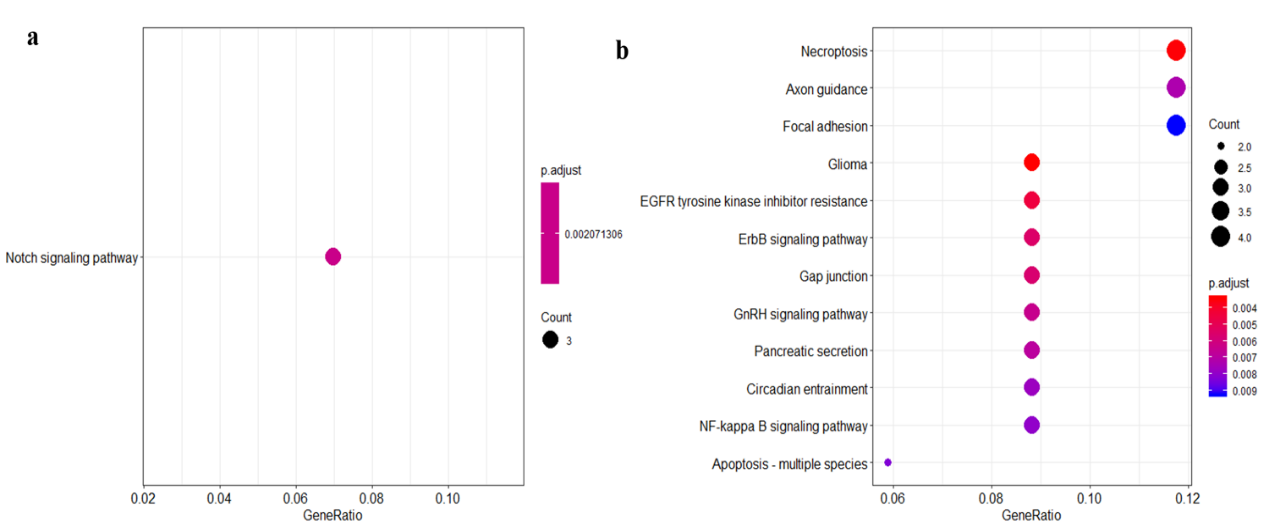


**Figure S2. Enriched developmental processes of consistently increasing methylation (IncrmCs) and consistently decreasing methylation (DecrmCs) in CpGs in ovarian methylomes across Pre-, In- and Post-puberty**. (**a**) The enriched KEGG pathways of IncrmCs. (**b**) The enriched KEGG pathways of DecrmCs. The IncrmCs were annotated as CpGs whose methylation level increased by $\geq$20% from Pre- to In-puberty and $\geq$20% from In- to Post-puberty; The DecrmCs were annotated as CpGs whose methylation level decreased by $\geq$20% from Pre- to In-puberty and $\geq$20% from In- to Post-puberty.


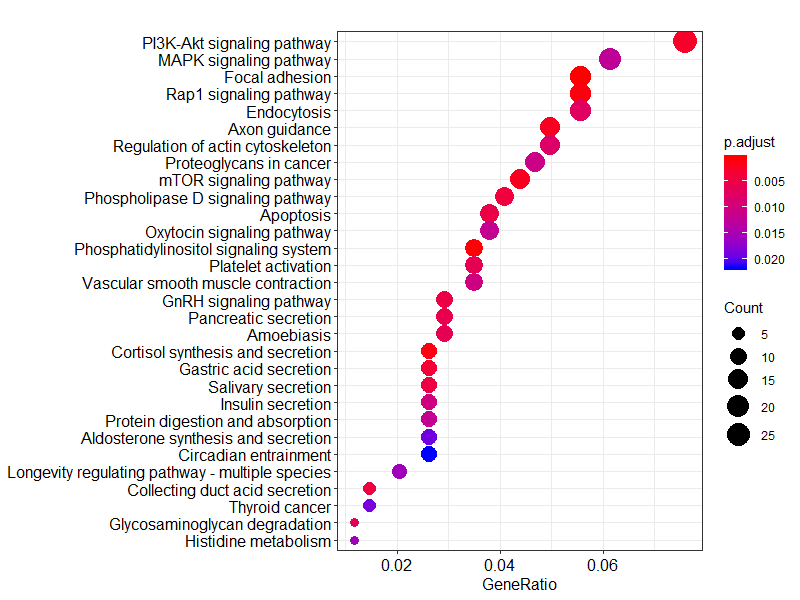


**Figure S3. Biological processes enriched with differentially methylated region genes.** The differentially methylated region genes were the genes that exhibited at least one DMR.
